# Enzymatic and Chemical Cross-Linking of Bacterial Cellulose/Fish Collagen Composites—A Comparative Study

**DOI:** 10.3390/ijms22073346

**Published:** 2021-03-25

**Authors:** Agata Sommer, Paulina Dederko-Kantowicz, Hanna Staroszczyk, Sławomir Sommer, Marek Michalec

**Affiliations:** 1 Department of Chemistry, Technology and Biotechnology of Food, Chemical Faculty, Gdańsk University of Technology, Narutowicza 11/12 St., 80-233 Gdańsk, Poland; agata.sommer@pg.edu.pl (A.S.); p.dederko@ihar.edu.pl (P.D.-K.); 2 Laboratory of Molecular Diagnostics and Biochemistry, Plant Breeding and Acclimatization Institute—National Research Institute, Bonin Research Center, Bonin 3, 76-009 Bonin, Poland; 3 Department of Automotive Engineering, Faculty of Mechanical Engineering and Ship Technology, Gdańsk University of Technology, Narutowicza 11/12 St., 80-233 Gdańsk, Poland; slawomir.sommer@pg.edu.pl; 4 Faculty of Chemistry, Jagiellonian University, Gronostajowa 2, 30-387 Kraków, Poland; michalec@chemia.uj.edu.pl

**Keywords:** bacterial cellulose, collagen, *ex situ* modification, cross-linking, structural characteristics

## Abstract

This article compares the properties of bacterial cellulose/fish collagen composites (BC/Col) after enzymatic and chemical cross-linking. In our methodology, two transglutaminases are used for enzymatic cross-linking—one recommended for the meat and the other proposed for the fish industry—and pre-oxidated BC (oxBC) is used for chemical cross-linking. The structure of the obtained composites is characterized by scanning electron microscopy, thermogravimetric analysis, X-ray diffraction, and Fourier transform infrared spectroscopy, and their functional properties by mechanical and water barrier tests. While polymer chains in uncross-linked BC/Col are intertwined by H-bonds, new covalent bonds in enzymatically cross-linked ones are formed—resulting in increased thermal stability and crystallinity of the material. The C2–C3 bonds cleavage in D-glucose units, due to BC oxidation, cause secondary alcohol groups to vanish in favor of the carbonyl groups’ formation, thus reducing the number of H-bonded OHs. Thermal stability and crystallinity of oxBC/Col remain lower than those of BC/Col. The BC/Col formation did not affect tensile strength and water vapor permeability of BC, but enzymatic cross-linking with TG_GS_ improved them significantly.

## 1. Introduction

Bacterial cellulose (BC) is a linear β-(1→4)-D-glucose polysaccharide synthesized by aerobic non-pathogenic bacteria of the genus: *Agrobacterium*, *Pseudomonas*, *Sarcina*, *Rhizobium*, and *Komagataeibacter* (formerly *Acetobacter*) [1]. Due to the high synthesis efficiency, the *Komagataeibacter xylinus* species is the most commonly used for BC production in a laboratory setting. During the synthesis, single glucose chains, produced inside the bacterial body, extrude out through the pores present on their cell envelope; then, in the hierarchical crystallization process, they form subfibrils, and then microfibrils, which finally bound to ribbons. The latter form a BC three-dimensional network structure, and numerous spaces between the fibers result in its highly porous surface [2,3]. The unique structure of BC leads to its remarkable properties, in both wet and dried forms [4,5]. This polysaccharide is characterized by high chemical purity and crystallinity, as well as high mechanical strength and water absorption properties [4,6,7]. Due to these features and because of high biodegradability and biocompatibility, BC-based materials have gained interest in many industries, especially the medical, pharmaceutical, and food industries. As the BC has a low biological activity, it is often found in combination with protein in these materials.

Collagen is the major structural protein of most connective tissues, including skin, bone, and tendons. It shows many important biological functions, such as tissue formation and cell adhesion, with excellent biocompatibility, low immunogenicity, and good film-forming properties. All these features give collagen a great potential in preparing scaffolds as a dural substitute, dressings, as well as edible sausage casings [8]. Due to the good barriers against oxygen and aromas, collagen can also be used to prepare biodegradable packaging materials [9]. Although this protein is mainly obtained from the skins or bones of porcine and cattle, its new sources are being sought because of diseases, such as bovine spongiform encephalopathy and transmissible spongiform encephalopathy. A rich source of cheap collagen is waste from the fish industry, which is burdensome for the environment due to the lack of rational management. Because of the lower cross-linking of fish collagen compared to that derived from mammalian tissues, the former is obtained under milder conditions. Colorless and odorless collagen can be isolated from fish skins by direct extraction with organic acid [10]. Nevertheless, fish collagen films have some disadvantages in physicochemical properties, such as, for example, low thermostability, and relatively poor mechanical properties, which limit their use. To overcome these problems, various efforts are made, including the blending of collagen with other polymers with different structures and its chemical and enzymatic treatments.

The BC-based composites can be formed either at the stage of BC synthesis, by incorporating the modifying component into the medium applied, *in situ method*, or by immersing the prepared BC membrane in a modifying component solution, *ex situ method*. It is difficult to obtain BC-collagen composites by *in situ* method. First, the bacteria present in the medium can use collagen as an additional source of carbon and nitrogen [11]. Second, the cleaning of the resulting composites and their sterilization can contribute to protein denaturation [12,13]. Third, a big collagen molecule can fall to the bottom of the culture dish and not penetrate the cellulose membrane that forms on the surface of the culture medium. As a result of these effects, the composites obtained do not have desired properties [3,6]. The *ex situ* method of obtaining BC/collagen composites may also be ineffective. According to Cheng et al. [12] and Shah et al. [3], the small sizes of the pores in the BC membrane can prevent the penetration of collagen molecules and their incorporation into the membrane in an amount sufficient to change the membrane properties. However, cross-linking of protein can contribute to enhancing its structure and eliminate the problem of its desorption from the polysaccharide matrix [7]. The generation of cross-linking bonds can be induced by a physical process, e.g., by applying heat or mechanical agitation, a chemical process by the addition of chemical cross-linkers, and an enzymatic process by enzyme catalysis [14]. Ionic, hydrophobic, and van der Waals interactions can take part in the cross-linking process, as well as covalent bonds can form. Chemical cross-linking compounds, e.g., aldehydes (glutaraldehyde, polyepoxides), are included in a formed bond, hence, are toxic [15]. 1-Ethyl-3-(3-dimethylaminopropyl) carbodiimide (EDC)—an agent commonly used to cross-link proteins—is not included in a formed bond and is transformed into water-soluble urea derivatives, which do not reveal toxicity. However, some amount of unreacted EDC can remain in the material and be toxic. Therefore, according to Ulubayram et al. [16], gelatin sponges modified with EDC immersed in a culture of fibroblasts resulted in a 90% decay of the cells. Thus, due to the absence of possible toxic effects, enzymatic catalysis is a safer process of protein cross-linking than chemical cross-linking. The agent which is most commonly used for enzymatic cross-linking is transglutaminase (TGase) [17]. This transferase catalyzes acyl-transfer reactions between γ-carboxyamide groups of glutamine residues and the ε-amino group of lysines in proteins, leading to inter- or intramolecular cross-linking. TGase has been found in animal and plant tissues, as well as in bacteria [18]. There are different microbiologically derived enzymatic preparations on the market which contain TGase obtained in a biosynthesis-dependent manner via *Streptoverticillium* sp. strain. Although they are recommended for various applications, including TGase for cross-linking of protein of meat, fish, and milk [19], there is little information on the details of their commercial production process and procedures applied.

This study aims to form BC composites with fish collagen by the ex situ method, and then to cross-link them with TGase. Two different TGase preparations were used to improve mechanical and water-vapor barrier properties of the materials obtained: One recommended for the meat and the other proposed for the fish industry. According to our knowledge, the effectiveness of cross-linking of two-component composites using various enzymatic preparations has not been compared yet. To study the effectiveness of the enzymatic cross-linking, chemical cross-linking was also performed using sodium periodate to oxidize the BC membrane [12]. It is well-known that the aldehyde groups, formed in the polysaccharide chains (in this case BC) during its oxidation, reacting with amino groups, found in the protein chains (in this case collagen), form a Schiff base. The covalent bonds formed in this base increase the strength of interaction between the composite components, when compared with the strength of the mainly electrostatic interactions that mostly occur between polysaccharides and proteins [6,12,20,21]. Finally, the changes in the BC structure that occurred due to the modifications applied were evaluated, and the effect of these changes on the mechanical and water barrier properties was examined. Thus, the presented results show the structure–property relationship of composites of bacterial cellulose and fish collagen after their enzymatic versus chemical cross-linking.

## 2. Results and Discussion

### 2.1. Composites Preparation

BC/fish collagen (BC/Col) composites were prepared by immersing wet BC membranes in the Col solution. To enhance the interaction between polymers and to eliminate the problem of protein desorption from the polysaccharide matrix, the composites were cross-linked with TGase. Two preparations were used for this purpose, one recommended for the meat industry (TG_WM_) and the other for the fish industry (TG_GS_), and their cross-linking efficiency was compared. The enzymatic reaction was running when the BC/Col composites were immersed in the TG_WM_ or TG_GS_ solution and shaken for 15 or 120 min. To obtain composites of oxidized BC (oxBC) with Col (oxBC/Col), the same procedure was used as that for the preparation of BC/Col, but the BC was pre-modified with sodium periodate oxidation according to the method described by Cheng et al. [12]. All resulting materials were covered with linen fabric to prevent their deformation and allowed to dry at room temperature. The average thickness of these materials ranged from 0.030 to 0.060 mm. To determine the effectiveness of the modification with Col, the protein content in the composites was determined by the Kjeldahl method (PN-75/A-04018) using a nitrogen-protein conversion factor of 6.77.

The total protein content in the BC/Col and oxBC/Col dried to constant weight was 12.81 ± 0.06% and 9.32 ± 0.04%, respectively. These values clearly prove the effectiveness of the modification of both BC and oxBC with Col by the ex situ method, although Cheng et al. [12] reported the ineffectiveness of this method in the case of BC. According to those authors, oxidation of the cellulose with NaIO_4_ allows the protein to be incorporated into the BC matrix with increased efficiency, due to the covalent bonds between the aldehyde groups of oxBC and the amine groups of the Col that are formed in the Schiff reaction. The results obtained do not fully support this statement, as the oxBC/Col composites contained about 20% less protein than the BC/Col. It seems that due to the BC oxidation, not only aldehyde groups are formed, but carboxyl groups as well, which with amino groups of protein form dissociating salts in an aqueous medium.

### 2.2. Structural Characteristics

#### 2.2.1. Morphological Properties

The surface and cross-section morphology of freeze-dried BC, oxBC, and their composites with Col were examined using scanning electron microscopy (SEM) using conditions enabling the testing of non-conducting materials. To observe the cross-sections, the samples were glued with carbon tape to a carbon steel cube.

**Figure 1:**
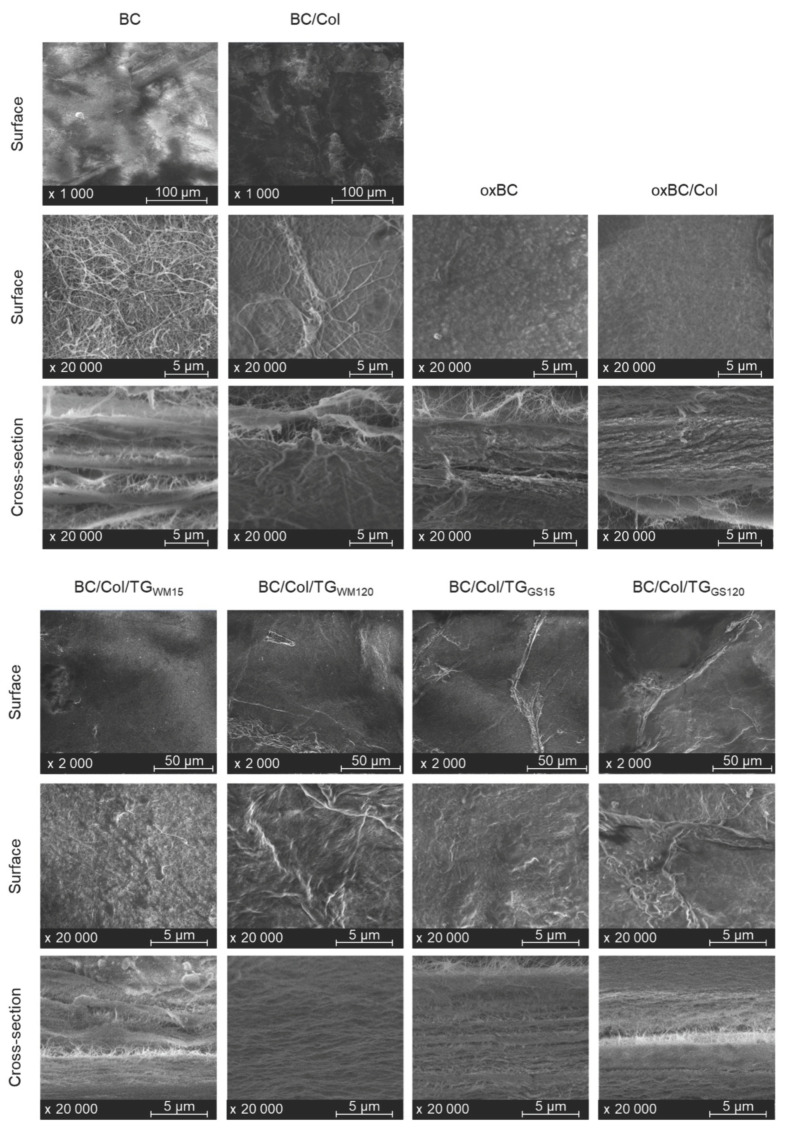
SEM images of bacterial cellulose (BC), oxidized BC (oxBC), and their composites with Col: BC/Col/TG_WM15_ and BC/Col/TG_WM120_–BC/Col cross-linked with TG_WM_ for 15 and 120 min, respectively; BC/Col/TG_GS15_, and BC/Col/TG_GS120_–BC/Col cross-linked with TG_GS_ for 15 and 120 min, respectively.

The SEM image of BC revealed a coherent, three-dimensional (3D) network of long cellulose fibers that formed a uniform surface with irregularly distributed pores, and a well-organized layered structure (Figure 1). As reported by Zhijiang and Guang [22], a high ratio of the fiber length to its diameter, i.e., the high aspect ratio, determines good mechanical properties of BC, especially its high resistance to tensile.

The SEM images of BC and BC/Col, taken at 1000× magnification, showed the smooth surface, while the surface of enzymatically cross-linked BC/Col composites was highly wrinkled. The surface of composites cross-linked for a shorter period of time was less wrinkled than that cross-linked for a longer period of time, and the surface of composites cross-linked with TG_GS_ was more wrinkled than that cross-linked with TG_WM_. The higher degree of wrinkling of the surface of BC/Col cross-linked with TG_GS_ than with TG_WM_ could indicate that the higher compressive stresses are generated in the former. These compressive stresses could increase the tensile strength as the forces applied to the material must first overcome these stresses before they cause the cellulose fibers to stretch. In turn, the SEM images, taken at 20,000× magnification, revealed the porous structure of both BC and BC/Col, with a similar pore diameter, ranged from 90 to 100 nm. The structure of BC/Col cross-linked with TG_WM_ and TG_GS_ also appeared to be porous, but with a much smaller pore size, below 70 nm. Likely, the smaller pores and the higher density of BC/Col cross-linked with TG_GS_ than that of uncross-linked one make it more difficult for water to penetrate the former, and consequently, to cause an increase in their resistance to tensile. The BC/Col cross-linked with TG_GS_ for 120 min was characterized by the wrinkled surface suggesting the presence of compressive stress, and this probably also impeded the water penetration.

The cross-section of the BC and BC/Col showed a layered structure of the material. However, the cellulose fibers visible between these layers in BC/Bol were more aggregated compared to those of BC, and their diameter was higher. The fiber diameter of BC was 40–50 nm, whereas those of BC/Col ranged between 150–180 nm. The higher diameter of fibers in the BC/Col indicates that they were covered with Col, which could prevent them from stretching. After the cross-linking of BC/Col with both TG_WM_ and TG_GS_, the layers came even closer to each other. According to Wangtueai et al. [23], the increased fiber thickness, and according to Elango et al. [24] and Liu et al. [25], the more compact layers, both indicate that cross-linking of the material takes place.

As a result of oxidation, the BC membranes shrank; however, the 3D structure of BC was preserved. The diameter of pores was reduced to about 30–50 nm, and the diameters of individual fibers were so small that the fibers were hardly visible, and the surface became smoother. The layers of oxBC were closer to each other than those of BC, as the cross-section image revealed. Cheng et al. [12] and Luo et al. [11] reported that the oxBC composites with Col differ in morphology depending on the amount of incorporated protein. When Col was incorporated into the oxBC matrix, its pore diameter did not change, although the diameter of the fibers increased enough to be distinguishable (20–30 nm). This made the surface even smoother. Moreover, the oxBC/Col fibers were highly aggregated. Cheng et al. [12] and Luo et al. [11] reported that the oxBC composites with Col differ in morphology depending on the amount of incorporated protein.

#### 2.2.2. Thermal Properties

The thermal stability of BC, oxBC, and their composites with Col was assessed by thermogravimetric analysis. The thermogravimetric (TG) and differential thermogravimetric (DTG) curves were used to determine the weight loss in the selected temperature ranges and the decomposition temperature of the material tested, respectively.

**Figure 2:**
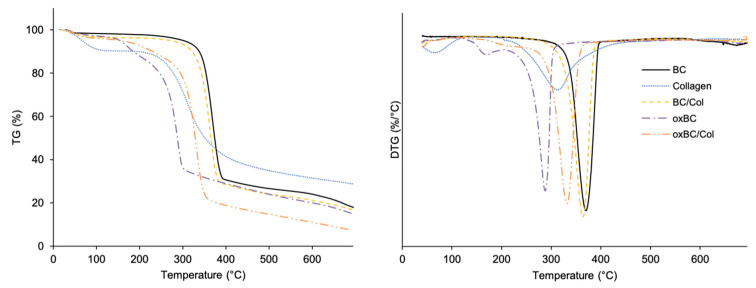
Thermograms of BC, oxBC, and their composites with Col: BC/Col and oxBC/Col, respectively.

A fast, one-step decomposition of BC took place at 363 °C with the loss of 84% of its weight within the range of 200–400 °C (Figure 2, Table 1). The Col lost at temperatures up to 200 °C ca. 9% of its weight, and its decomposition occurred at 313 °C. The inclusion of protein into the BC structure did not significantly change the thermal stability of BC, as the BC/Col composite decomposed at 364 °C with 86% weight loss.

All thermograms of BC/Col cross-linked with TGase, both TG_WM_ and TG_GS_, resembled that of uncross-linked one. Their decomposition temperatures were similar; however, their weight losses were 14–16% lower than that of uncross-linked BC/Col (Table 1). Thus, it seems that the thermal stability of BC/Col increases, due to enzymatic cross-linking. Because the lowest weight loss was noted for BC/Col cross-linked with TG_GS_, the preparation proposed for the fish industry seems to be a more effective fish collagen cross-linker than TG_WM_ than the preparation recommended for the meat industry. Furthermore, the increase in thermal stability could result not only from the formation of cross-linking bonds in the composite, but also from an increase in its crystallinity degree [25,26,27].

Decomposition temperature of oxBC was 75 °C lower than that of BC (Table 1). Moreover, the oxBC thermogram showed an additional endothermic peak at 172 °C, which in the BC thermogram was not observed. Apparently, oxBC lost a significant amount of water at temperatures up to 200 °C. It seems that the destruction of the internal structure of BS, which occurs as a result of the oxidation process, reduces the degree of BC water-mediated cross-linking, thus releasing water molecules. According to Cheng et al. [12], the lower cross-linking degree of BC decreases its thermal stability. The incorporation of Col into oxBC fibers increased its thermal stability. Although the decomposition temperature increased by 44 °C in relation to oxBC (Table 2), it was still 30 °C lower than BC and BC/Col.

#### 2.2.3. Crystalline Properties

**Figure 3:**
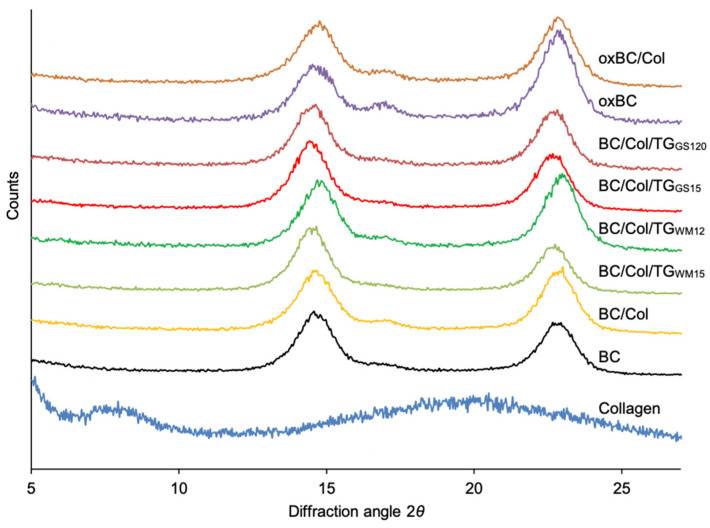
XRD diffractograms of BC, oxBC, and their composites with Col: BC/Col/TG_WM15_ and BC/Col/TG_WM120_–BC/Col cross-linked with TG_WM_ for 15 and 120 min, respectively; BC/Col/TG_GS15_, and BC/Col/TG_GS120_–BC/Col cross-linked with TG_GS_ for 15 and 120 min, respectively.

X-ray diffraction (XRD) diffractograms of BC, oxBC, and their composites with Col showed three peaks with a maximum at 14.6°, 16.2° and 22.6° 2 *θ* (Figure 3), which are attributed to 110, 110, and 200 crystal planes of cellulose, respectively [21,22]. According to Bragg’s law, the identity distance (*d*) of each crystal plane is 6.06 (*d_1_*), 5.52 (*d_2_*), and 3.92 nm (*d_3_*) (Table 2), which is assigned to the typical cellulose I crystal [21,22]. The diffractogram of Col indicated that it was an amorphous material; however, the main peaks at 7.7 and 20.2° 2 *θ* were distinguished [28,29,30]. The former peak is related to the diameter of the triple helix and the latter to the distance between amino acidic residue along this helix.

As no differences were found between the diffraction patterns of BC and BC/Col, and between the uncross-linked and enzymatic cross-linked BC/Col, the results of the XRD analysis confirm the findings of the SEM (Figure 1) and thermal (Table 1) analysis that the BC structure does not change after the Col introduction, and after the enzymatic cross-linking of the resulting composites changes only slightly. On the other hand, differences in the diffraction patterns of BC and oxBC indicated a change in the native order and crystalline structure of BC, due to the oxidation process, as the significant increase in the intensity of the peak at 16.6° 2 *θ* in the oxBC pattern was observed. According to Ul-Islam et al. [31], this peak corresponds to crystal form Iγ, which is believed to be an intermediate form between I_α_ and I_β_, but more like I_α_.

Despite the lack of clear differences in diffraction patterns of BC and BC/Col, there was a slight shift of the peak centered at 22.6° 2 *θ* in the BC pattern towards higher diffraction angles in the BC/Col pattern (Figure 2). As Table 2 revealed, this shift was due to a decrease in *d_3_* in the crystal form of BC, from 3.92 to 3.86 nm. In turn, the peak appearing at 7.7° 2 *θ* in the Col pattern, indicating that the distance between the triple helix chains of Col fibrils was 1.15 nm, as calculated by Bragg’s law, was not visible in the BC/Col pattern. It could be due to two factors. First, there was a small amount of protein in BC/Col, but this seems to be contrary to the results of the analysis for the protein content in the composite. Second, the Col structure changed, and the BC structure did not change when the BC/Col was formed. It is well known that the polysaccharide chains formed during BC synthesis create hydrogen bonds that define the crystalline structure of their membranes, which is difficult to change by ex situ chemical modification [32]. It can, therefore, be assumed that the BC modification with Col leads to the alignment of the protein fibrils between the cellulose fibrils, which is why the peak attributed to the triple helix diameter is not visible in the BC/Col patterns.

The crystallinity index (CrI) of BC was 86% (Table 2). After modification with Col, the CrI slightly decreased, after cross-linking of BC/Col with TG_WM_ remained at the same level, while increased again after cross-linking with TG_GS_. Thus, it seems that the increase in thermal stability of BC/Col cross-linked with TG_GS_ (Table 1) was due to the increase in its crystallinity degree, indeed. According to Ul-Islam et al. [31], the BC modification with another polymer usually leads to decreased crystallinity, which can result from both a lower crystallinity of the introduced polymer and interference in the structure of regularly arranged BC fibers [22]. The high CrI of the BC/Col cross-linked with TG_GS_, even higher than the CrI of BC, clearly indicates an increased number of cross-linking bonds in the composite as a result of using this preparation.

The BC oxidation decreased the CrI by ca. 8%. After the protein introduction into oxBC matrix the CrI increased again; however, that of BC and BC/Col was no longer achieved. The cleavage of the C2-C3 bond in the D-glucose units of BC causes not only the formation of aldehyde groups, but also reduces the number of hydrogen bonds between OH groups present in the polysaccharide chains. Destruction of the order of these chains leads to the reduction in the BC crystallinity [20,21]. In turn, the increase in the CrI caused by the inclusion of Col into the oxBC matrix probably results from interactions between both polymers.

#### 2.2.4. Chemical Structure

To identify chemical structural properties of all materials obtained, Fourier transform infrared spectroscopy (FT-IR) technique was applied.

Table 3 presents the FT-IR spectral characteristics of BC and Col [4,6,31,33,34,35,36,37,38]. To verify the integrity of the triple helix in the protein structure, the absorbance ratio A_1228_/A_1441_ in its spectrum was calculated. This ratio exceeded unity (Table 2), which proves that the secondary structure in the Col extracted was preserved [35,36].

**Figure 4:**
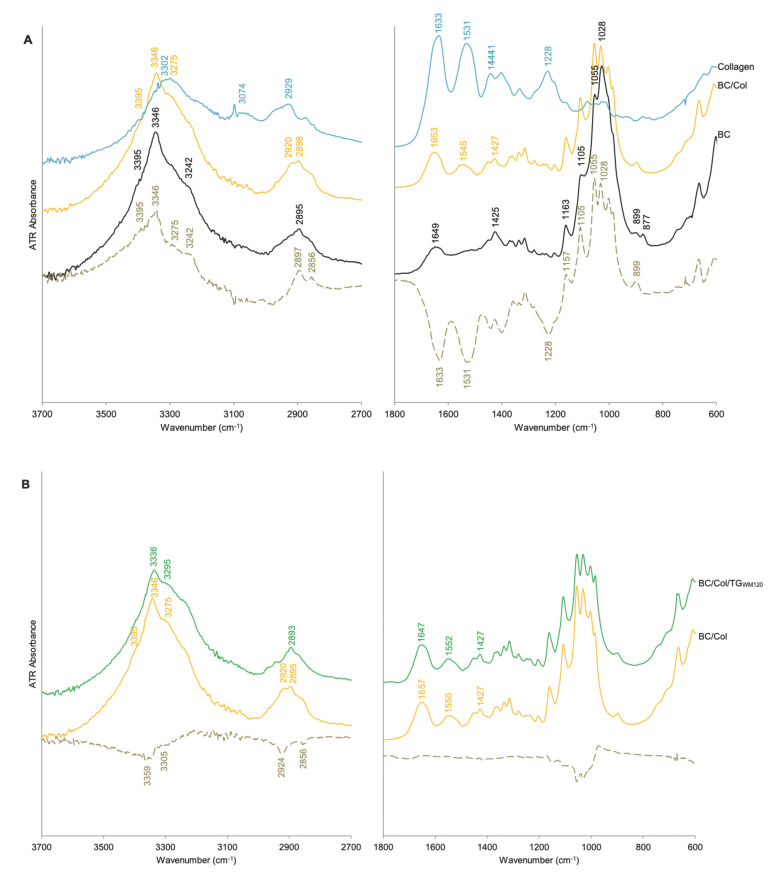

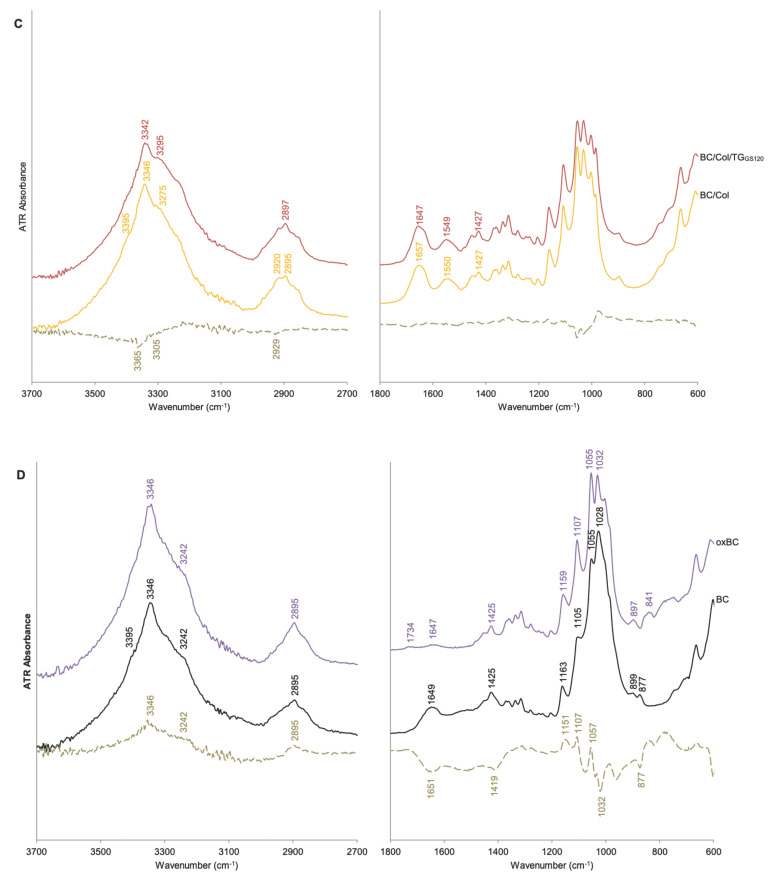

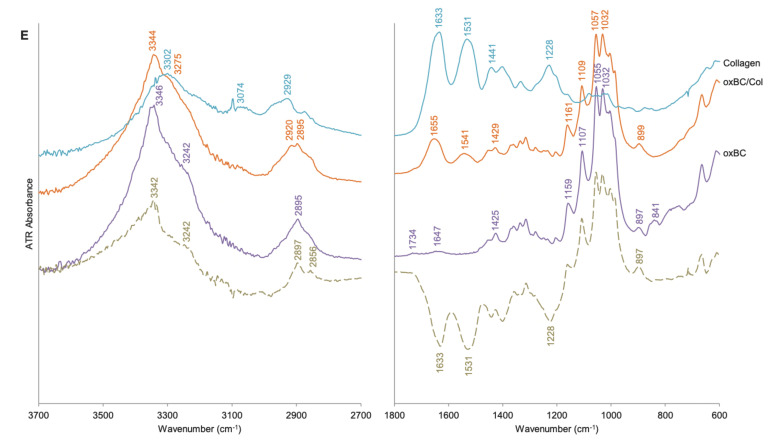
FT-IR spectra ( ) of BC, oxBC, and their composites with Col: BC/Col/TG_WM120_ and BC/Col/TG_GS120_–BC/Col cross-linked for 120 min with TG_WM_ and TG_GS_, respectively. Differential spectrum ( ) of (**A**) BC/Col from which the spectrum of BC was subtracted; (**B**) BC/Col/TG_WM120_ from which the spectrum of BC/Col was subtracted; (**C**) BC/Col/TG_GS120_ from which the spectrum of BC/Col was subtracted; (**D**) oxBC from which the spectrum of BC was subtracted; (**E**) oxBC/Col from which the spectrum of oxBC was subtracted.

**Figure 5:**
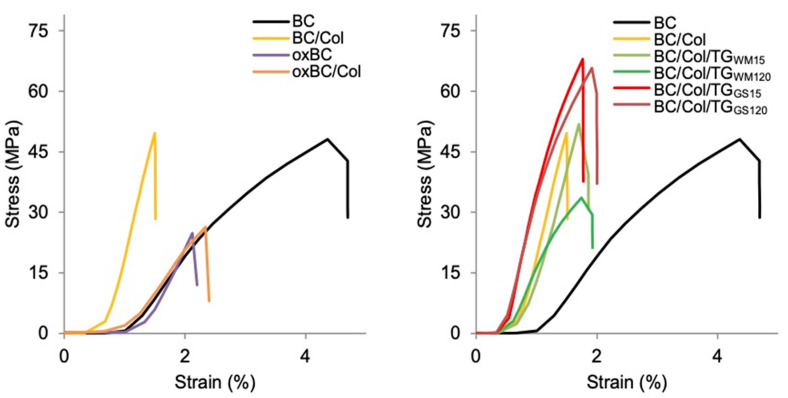
Stress-strain curves of BC, oxBC, and their composites with Col.

Although the FT-IR spectra of the BC- and oxBC-based composites indicated that cellulose is their dominant component (Figure 4), clearly distinguished amide I and amide II bands proved the presence of protein in them. These bands were not observed in the BC and oxBC spectra, but were evident in the Col spectrum; however, at different wavenumbers. The amide I and amide II bands in the Col spectrum observed at 1633 and 1531 cm^−1^ (Table 3) were shifted in the spectra of composites by ca. 20 and 10 cm^−1^, respectively, toward higher wavenumbers (Figure 4A,E). Apparently, the energy of interaction involving –C=O, –NH, and –CN groups increased as the formation of the BC/Col and oxBC/Col required conformational changes of protein to ensure the composite formation. An essential change in the shape of the complex band at the 2950–2800 cm^−1^ range, associated with the stretching vibrations of the C–H (ν_CH_), indicates changes in the arrangement of polymer chains after BC modification with Col, seems to confirm this hypothesis. In contrast, the negative peaks at 1633 and 1531 cm^−1^ in the differential spectra of BC/Col (Figure 4A) and oxBC/Col (Figure 4E) appearing at exactly the same wavenumbers as the amide I and amide II bands in the Col spectrum, pointed out that not all protein was incorporated into composites. Further, an increase in the band intensity in the range of 3600–3000 cm^−1^, due to the ν_OH_ characteristic for both BC (oxBC) and Col, and the ν_NH_ typical only of protein, was also observed. These changes were accompanied by the appearance of the peak at 3346 cm^−1^ in the difference spectra of BC/Col (Figure 4A) and oxBC/Col (Figure 5E) with shoulders at 3275 and 3242 cm^−1^. While the first and third maxima were due to the H-bonded ν_OH_, intra- and intermolecular, respectively (Table 3) and indicated the presence of BC in the composite, the second maximum confirmed the Col presence, as it was due to the ν_NH_. The shift of the latter by 30 cm^−1^ toward lower wavenumbers is indicative of the formation of hydrogen bonds between the functional groups of the polysaccharide and protein.

The formation of BC/Col had no effect on the position and intensity of the bands at 1430 and 1360 cm^−1^, referred to BC ‘crystalline’ absorption bands [33,37], while it led to the decrease in the A_1228_/A_1441_ ratio (Table 2), assigned to the Col triple helix structure. These observations confirm once again the results of XRD analysis that the ex situ modification of BC with Col does not affect the crystalline structure of BC, but changes the structure of the protein triple helix.

The FT-IR spectra proved that the TGase leads to the cross-linking of BC/Col composites, no matter which preparation (TG_WM_ or TG_GS_) was applied and what the cross-linking time was (15 or 120 min). The spectra of the enzymatic cross-linked composites showed a decrease in the intensity of the amide A and amide II bands, and an increase in the intensity of the amide I band. These changes were confirmed by the negative peak observed at 3305 cm^−1^ in the difference spectra, and by the decreasing A_Amide A_/A_Amide I_ ratio (Table 2). Since the formation of the covalent bonds between the ε-amine group of a lysine residue and the γ-carbonyl group of glutamine residues in the presence of TGase results in the formation of iso-peptide bonds [39], the number of bounded NH groups increases as the cross-linking progress. The amide A and amide II bands are characteristic of NH_2_ groups, and the amide I band is characteristic of peptide bonds (Table 4); thus, the decreasing intensity of the former indicates the change of free –NH_2_ groups into –NH groups, while the increasing intensity of the latter points out the new isopeptide covalent bond formation. This observed trend is in line with previous findings [35,38,40]. The calculated A_Amide A_/A_Amide I_ ratio was also used to determine the cross-linking efficiency. As the lower value of this ratio, the higher the cross-linking degree of the composite, the values listed in Table 3 indicate that the composites cross-linked with TG_GS_ were characterized by the highest cross-linking degree.

Although the oxBC spectrum seemed to cover the BC spectrum completely (Figure 4D), some details manifested differences in the BC structure, due to its oxidation. The fine bands observed at 1734 cm^−1^ and at 841 cm^−1^ in the oxBC spectrum, corresponding to the carbonyl groups and hemiacetal moieties, respectively [6,12,20,21], indicated mild oxidation of BC. Moreover, an increase in the intensity at 1163, 1055, and 1105 cm^−1^, corresponding to the ν_C–O_ and the ν_C–H_, respectively, and a decrease in the intensity at 1032 cm^−1^, corresponding to the δ_C–O–H_, confirmed by positive peaks at 1151, 1057 and 1107 cm^−1^, and negative peak at 1032 cm^−1^ in the oxBC differential spectrum, indicated the vanishing of secondary alcohol groups in favor of the carbonyl groups formation. Likely, the change of the hydroxyl groups into the carbonyl groups reduced the degree of BC water-mediated hydrogen bonding. All these changes proved the oxidation of vicinal diols. In turn, the vanishing of the bands at 1734 and 841 cm^−1^ in the oxBC/Col spectrum confirmed the reaction between the carbonyl groups of oxBC and free –NH_2_ groups of the Col [12].

### 2.3. Functional Properties

#### 2.3.1. Mechanical Properties

Tensile specimens were cut off in the form of rectangular strips 15 × 100 mm in size and tested on a universal testing machine, generating the stress–strain curve for each of them (Figure 5). Fourteen specimens were tested for each sample, and the average values of the tensile strength (σ) and elongation at break (ε) were reported (Table 4).

The tensile tests revealed that the mechanical properties of the materials tested are largely due to their structure. As the thermal stability expressed by the weight loss and decomposition temperature (Table 1), as well as the crystalline structure expressed by the CrI (Table 2), did not change after the Col introduction into the BC matrix, and the chains of both polymers were intertwined only by weak hydrogen bonds as the analysis of the FTIR spectra showed (Figure 4A), the immersion of BC in the Col solution did not significantly affect the σ value. However, it decreased the ε value almost 3-fold (Table 4). According to Saska et al. [13], the tensile strength of composites of BC with Col is lower, but according to Zhijiang and Guang [22], Albu et al. [41], and Yang et al. [4], it is higher than that of BC. However, Saska et al. [13] modified BC with rat collagen, Zhijiang and Guang [22] with pork collagen, and Albu et al. [41] with bovine collagen, and it is well-known that fish collagen differs from a mammalian counterpart in the intrinsic properties, including an individual amino acids content [42]. Moreover, collagen from cold-water fish species is different from that from warm-water species. The reduction in ε, due to BC modification with gelatin isolated from calfskin, was reported by Nakayama et al. [43]. According to Zhijiang and Guang [22], the decrease in the ε value of BC composites with Col arises from the inclusion of the protein into the BC pores, preventing cellulose fibers from stretching.

The results of mechanical tests also confirmed the previous suppositions, that the highly wrinkled surface of enzymatically cross-linked BC/Col composites can increase their tensile strength. The highest σ value was found for the BC/Col composites cross-linked with TG_GS_, the surface of which showed the highest degree of wrinkling (Figure 1). These composites also showed the highest thermal stability (Table 1), which in turn resulted from their highest CrI and the structure strongly cross-linked with covalent bonds, expressed by the lowest A_Amide A_/A_Amide I_ ratio (Table 2). At the same time, enzymatic cross-linking did not have a significant effect on the ε value, although a tendency of a slight increase in ε under the prolongation of cross-linking time could be observed. There is no information in the available literature on the effect of enzymatic cross-linking of BC/Col on the values of σ and ε. Although the data regarding the cross-linking of other proteins and protein-polysaccharide composites by using TGase indicate that such cross-linking tends to increase the σ values and decrease the ε value [44,45], the data reporting the opposite effect can also be found. However, the literature data show that the cross-linking effect is influenced by the type of TGase preparation used for this purpose. For example, cross-linking of the bovine gelatin-based film with TGase dedicated for the meat industry applications improved σ [46], while cross-linking of fish gelatin-based films with this TGase did not affect σ values [47].

The reduced thermal stability of oxBC in relation to BC, resulting from the destruction of the native order and crystalline structure of BC, affected the mechanical parameters. The oxidation of BC decreased both σ and ε almost 2- and 3-fold, respectively. As shown by the analysis of FT-IR spectra, the oxidation reaction caused not only the formation of aldehyde groups, but also a reduction in the number of hydrogen bonds between OH groups present in the BC chains. Moreover, as reported by Cheng et al. [12], the oxidation of BC with NaIO_4_ destroys its native structure, reduces its polymerization degree, and makes its mechanical properties worse, due to undesirable side reactions, called peeling reactions. Therefore, to limit the adverse effect of these reactions, the authors reduced the oxidation time from 12 to 2 h. But even the oxidation time of 2 h did not prevent the reduction of σ and ε, as the values listed in Table 1 indicate. Modification of oxBC with Col did not significantly change the values of these parameters. Despite the formation of covalent bonds between the carbonyl groups of the oxBC and the free –NH_2_ groups of the Col, σ and ε were lower than those of BC (Table 4).

#### 2.3.2. Water Vapor Permeability

Moisture management of BC, oxBC, and their composites with Col was assessed through the measurement of water vapor permeability (WVP) according to the ASTM method E 96–95 [48]. Three specimens were tested for each sample, and the average value was reported (Table 4).

Neither modification of BC with Col, nor the enzymatic cross-linking of BC/Col, nor the oxidation of BC and oxBC/Col formation did significantly affect the WVP (Table 4). A significant reduction in WVP by ca. 35% was observed only in the case of BC/Col cross-linked with TG_GS_ for the period of 120 min. Apparently, the increased resistance to tensile of these composites caused by the high cross-linked structure with the new covalent bonds resulting in the increased compactness of the material, impeded the water penetration. Schmid et al. [49] noted an improvement in barrier properties in whey protein films cross-linked with TGase preparation that was dedicated to dairy products. In turn, no significant change in WVP of fish gelatin films cross-linked with TGase preparation intended for use in the meat industry was observed by Yi et al. [45]. Hence, it can be assumed that the improvement of water barrier properties, similarly to mechanical properties, is influenced by the type of cross-linking preparation.

Reduction in WVP of protein-based materials, due to their cross-linking, is usually caused by a decrease in both the mobility of polypeptide chains and the free space in the structure, due to the formation of new bonds [23,50]. Such a reduction in WVP of the cross-linked protein-based composites can also be associated with the transformation of primary amine groups into less hydrophilic, secondary amine groups. According to Di Pierro et al. [51], the presence of less hydrophilic groups in the composite hinders the diffusion of water vapor particles and improves its barrier properties.

## 3. Materials and Methods

### 3.1. Materials

BC produced according to the PL171,952 B1 [52], PL 2,122,003 B1 [53], and US 6,429,002 B1 [54] was supplied by BOWIL Biotech Sp. Z o.o. (Władysławo, Poland). Col was prepared from the skins of silver carp (*Hypophthalmichthys molitrix*) as described by Sadowska et al. [10]. Two commercial TGase preparations, ACTIVA^®^WM recommended for the meat industry (TG_WM_), and ACTIVA^®^GS dedicated to the fish industry (TG_GS_), were purchased from Ajinomoto, Tokyo, Japan. Apart the enzyme, the former contained 99% maltodextrins, and the latter—maltodextrins, sodium chloride, gelatin, sodium phosphate, and safflower oil. Sodium periodate was purchased from Merck (Warsaw, Poland), and all other chemical reagents were supplied by the Avantor Performance Materials (Gliwice, Poland).

### 3.2. Modification of BC

To prepare the BC/Col, Col was dissolved in a 1% (*v*/*v*) aqueous solution of acetic acid to achieve its final concentration of 1% (*w*/*v*), and then wet BC membranes of a water content up to 97.7 % were immersed in that solution for 24 h at 20 °C. After the immersion, the BC/Col obtained were washed with distilled water six times for 15 min each time.

Before the BC/Col were cross-linked, the preparation of TGase was mixed with cold distilled water in an ice bath for 3 min, and then, the protein content in the solution obtained was determined by Lowry method [55]. Next, the BC/Col were immersed in the TG_WM_ or TG_GS_ solution used at a concentration corresponding to 0.09 mg protein/mL, followed by shaking (150 rpm) for 15 or 120 min at room temperature. After the reaction was completed, cross-linked composites were washed with several portions of distilled water to remove the unreacted enzyme.

To obtain chemically cross-linked composites, the BC was pre-modified with sodium periodate oxidation, according to the method described by Cheng et al. [12]. Briefly, the wet BC membrane was immersed in 150 mL of the 3% (*w*/*v*) solution of NaIO_4_ and kept in the dark at 37 °C for 2 h under shaking condition (150 rpm). After the reaction was completed, the excess of NaIO_4_ was removed by repeated rinsing with distilled water, and the oxBC membranes was drained of excess water with filter paper. To obtain the oxBC/Col, the same procedure as that to obtain BC/Col was used.

### 3.3. Structural Characterization

#### 3.3.1. Scanning Electron Microscopy

The surface and cross-section of freeze-dried BC, oxBC, and their composites with Col were examined on a Versa 3D Dual Beam scanning electron microscope (FEI, Hillsboro, OR, USA) with an accelerating voltage of 5 kV and the current 1.6 or 3.3 pA.

#### 3.3.2. Thermal Analysis

To study the effect of BC modification on its thermal properties, thermogravimetric analysis was performed using TA Instrument SDT Q600 (New Castle, DE). The samples of 10–20 mg were heated in open aluminum pans under a dynamic nitrogen atmosphere within a temperature range of 40–700 °C, at a heating rate of 10 °C/min.

#### 3.3.3. X-ray Diffractometry

To study the crystal structure of materials obtained, their XRD diffracrograms were recorded in a Philips type X’Pert Pro diffractometer (Eindhoven, The Netherlands) by using Cu Kα radiation at 30 mA and 40 kV. The spectra over the range of 0–45.0° 2 *θ* were recorded at scan rate of 0.02° 2 *θ*/s.

The CrI materials were calculated by following the equation proposed by Segal et al. [56].

$$CrI=\frac{\left(I_{200}\;-I_{am}\right)}{I_{200}}\times \;100$$

where *I_200_* and *I_am_* are the maximum intensities of diffraction at 2 *θ =* 22.6 and 18°, respectively.

#### 3.3.4. Attenuated Total Reflectance (ATR) Fourier Transformation Infrared Spectroscopy

To evaluate the molecular structures of all materials, the FT-IR spectra of their samples were recorded on a Nicolet 8700 spectrophotometer (Thermo Electron Scientific Inc., Waltham, MA, USA), using a Golden Gate ATR accessory (Specac, Orpington, UK) equipped with a single-reflection diamond crystal. The temperature during measurements was kept at 25 ± 0.1 °C using an electronic temperature controller (Specac, Orpington, UK). One hundred twenty-eight scans were collected for each spectrum, with a resolution of 4 cm^−1^. The spectrometer’s EverGlo source was on turbo mode during measurements. The spectrometer and ATR accessory were purged with dry nitrogen to reduce water-vapor contamination of the spectra, and all samples were conditioned for seven days in desiccators over P_2_O_5_ to remove residual moisture. For each sample aliquot, three to five replicate spectra were recorded to assess precision and ensure the reproducibility of each sample.

### 3.4. Mechanical Tests

Tensile strength and elongation at break of all materials tested were determined according to the ASTM method D 882-00 [57] with a model 5543 Instron Universal Testing Machine (Instron Co., Canton, MA, USA). Initial grip separation and cross-head speed were set at 50 mm and 10 mm/min, respectively. Strips of dried at room temperature, and materials samples (15 × 100 mm) were conditioned for 48 h at 25 ± 2 °C and 50 ± 2% relative humidity (RH) before determination of σ and ε.

### 3.5. Water Vapor Permeability

Water vapor permeability of materials was determined according to the ASTM method E 96-95 [48]. Dried at room temperature materials were conditioned for 24 h at 25 ± 2 °C and 50 ± 2% RH. Film samples were mounted on cups filled with water, and then the cups were placed, at 25 ± 2 °C and 50 ± 2% RH, in a desiccator. The weight of the cups was measured at 1 h intervals during 9 h. Simple linear regression was used to estimate the slope of weight loss vs. time plot.

WVP was calculated from:

$$WVP\;=\frac{WVTR\times \;L}{∆p}$$

where water vapor transmission rate (WVTR) is the slope/film area (g/m^2^ × h), *L* is the film thickness (mm), and Δp is the partial water vapor pressure difference (kPa) between the two sides of the material.

### 3.6. Statistical Analysis

Results in tables are averages from three to ten replications ± standard deviation. Data were evaluated statistically by analysis of variance (one-way procedure) using SigmaPlot 11.0, (SYSTAT Software, Erkrath, Germany). Differences between the means were determined by the Holm-Sidak method (*p* < 0.05).

## 4. Conclusions

This study compared BC/Col composites, cross-linked by the enzymatic and chemical reactions. Σ, ε, and WVP values obtained indicate the higher effectiveness of the enzymatic over the chemical cross-linking. The effectiveness of the former depends on the choice of the enzyme preparation for the collagen used. BC composites with collagen extracted from silver carp skins cross-linked with TG_GS_, recommended for fish protein cross-linking, are characterized by higher σ and reduced WVP compared to those cross-linked with TG_WM_, advised to be used in the meat industry. These properties result from changes in the BC/Col structure. As the FT-IR spectra revealed, in uncross-linked BC/Col composites, the polymer chains are intertwined by hydrogen bonds that form between the polysaccharide and protein functional groups, while in enzymatically cross-linked composites, the new covalent isopeptide bonds are formed. The composites cross-linked with TG_GS_ are characterized by a higher cross-linking degree than those cross-linked with TG_WM_; therefore, the degree of crystallinity and thermal stability of the former is higher than the latter.

The BC oxidation lowers its σ, and the chemical cross-linking of oxBC with Col does not significantly change this value. However, as a result of the oxidation process, the native order of the BC structure is changed. The cleavage of the C2-C3 bonds in the D-glucose units of BC causes8 not only the vanishing of secondary alcohol groups in favor of the carbonyl groups formation, but also reduces the number of hydrogen bonds between OH groups present in the polysaccharide chains. These changes result in the reduction in polymer crystallinity and a decrease in its thermal stability. Although the thermal stability and degree of crystallinity increase, due to the reaction of the carbonyl groups of oxBC with free –NH_2_ groups of the Col, these parameters in the oxBC/Col composite remain lower than BC and BC/Col, due to the change in the native order of BC following the oxidation.

## Figures and Tables

**Table 1:** Thermal properties of BC, oxBC, and their composites with Col.

Sample *	Temperature Range [°C]	Weight Loss [%] **	DTG [°C]
BC	40–200 200–400 400–700	1.9 84.1 6.6	363.0
Col	40–200 200–400 400–700	8.8 48.4 10.0	313.1
BC/Col	40–200 200–400 400–700	1.3 86.3 6.3	363.8
BC/Col/TG_WM15_	40–200 200–400 400–700	2.3 74.4 6.8	365.7
BC/Col/TG_WM120_	40–200 200–400 400–700	2.2 75.1 6.3	364.0
BC/Col/TG_GS15_	40–200 200–400 400–700	2.5 74.4 5.6	364.5
BC/Col/TG_GS120_	40–200 200–400 400–700	2.6 72.3 7.0	364.0
oxBC	40–200 200–400 400–700	11.5 59.0 8.9	172.2 288.0
oxBC/Col	40–200 200–400 400–700	6.4 73.9 7.9	205.8 331.7

* BC/Col/TG_WM15_ and BC/Col/TG_WM120_–BC/Col cross-linked with TG_WM_ for 15 and 120 min, respectively; BC/Col/TG_GS15_, and BC/Col/TG_GS120_–BC/Col cross-linked with TG_GS_ for 15 and 120 min, respectively. ** Weight loss (%) in the specified temperature ranges.

**Table 2:** d-Spacing and crystallinity index (CrI) of BC, oxBC, and their composites with Col determined through XRD analysis, and the absorbance ratio at 1228 and 1441 cm^−1^, and amide A and amide I through FT-IR analysis.

Sample ^x^	d-Spacing (nm)			CrI (%)	A_1228_/A_1441_	A_Amide A_ /A_Amide I_
	110	110	200			
	*d_1_*	*d_2_*	*d_3_*			
Col	–	–	–	–	1.04	1.27
BC	6.06	5.52	3.92	86.0	–	–
BC/Col	6.05	5.22	3.86	85.7	0.93	1.83
BC/Col/TG_WM15_	6.03	5.41	3.90	85.8	0.97	1.36
BC/Col/TG_WM120_	6.01	5.43	3.86	85.3	0.97	1.28
BC/Col/TG_GS15_	6.13	5.43	3.92	87.5	1.01	0.96
BC/Col/TG_GS120_	6.05	5.32	3.89	86.2	0.97	0.66
oxBC	6.09	5.21	3.89	79.1	0.99	–
oxBC/Col	5.99	5.34	3.89	82.2	0.93	1.63

^x^ BC/Col/TG_WM15_ and BC/Col/TG_WM120_–BC/Col cross-linked with TG_WM_ for 15 and 120 min, respectively; BC/Col/TG_GS15_, and BC/Col/TG_GS120_–BC/Col cross-linked with TG_GS_ for 15 and 120 min, respectively.

**Table 3:** Band assignments in the FT-IR spectra of native BC and Col.

BC		Col	
Position in cm^−1^ and Intensity *	Band Assignment	Position in cm^−1^ and Intensity *	Band Assignment
3395 sh	ν_OH_ intramolecular H-bonds for 3OH–O5 and 2OH–O6	3302 s	Amide A ν_NH_, ν_OH_
3346 s	ν_OH_ intramolecular H-bonds for 3OH–O5	3074 m	Amide B ν_NH_
3242 m	ν_OH_ intermolecular H-bonds for 6OH–O3′	2929 m	asym ν_CH2–_
2895 m	ν_CH_	1633 s	Amide I ν_C=O_, ν_NH_
1649 w	ν_C=O_, δ_OH_ polymer bound water	1531 s	Amide II δ_NH_, ν_C–N_, ν_C–C_
1425 w	δ_CH2_, δ_OH_	1441 m	δ_CH2_
1363 w	ν_CH_, δ_COO_	1228 m	Amide III ν_C–N_, δ_NH_
1163 w	δ_C–O–C_ of C1–O–C4, δ_OH_		
1105 m	ν_C–OH_ of C2–OH		
1055 vs	δ_C–OH_ of C3–OH		
1028 vs	ν_C–O_ of C6–OH		
1003 vs	ν_C–OH_		
999 vs	β-glycosidic linkage		
899 w	δ_CH2_		

* Abbreviations are related to band intensity—vs, very strong; s, strong; m, medium; w, weak; vw, very weak; sh, shoulder.

**Table 4:** Mechanical and water vapor properties of BC, oxBC, and their composites with Col.

Sample *	σ [MPa] **	ε [%] **	WVP [g∙mm∙kPa^−1^∙h^−1^∙m^−2^] ***
BC	48 ± 3 ^b^	4.1 ± 0.8 ^a^	0.083 ± 0.010 ^bc^
BC/Col	45 ± 7 ^b^	1.4 ± 0.5 ^c^	0.086 ± 0.007 ^bc^
oxBC	28 ± 8 ^c^	1.6 ± 0.4 ^bc^	0.075 ± 0.003 ^abc^
oxBC/Col	27 ± 3 ^c^	2.3 ± 0.5 ^b^	0.093 ± 0.019 ^c^
BC/Col /TG_WM15_	45 ± 9 ^b^	1.8 ± 0.6 ^bc^	0.066 ± 0.008 ^abc^
BC/Col /TG_WM120_	36 ± 4 ^c^	1.9 ± 0.6 ^bc^	0.061 ± 0.009 ^ab^
BC/Col /TG_GS15_	63 ± 9 ^a^	1.6 ± 0.3 ^bc^	0.074 ± 0.006 ^abc^
BC/Col /TG_GS120_	60 ± 7 ^a^	2.1 ± 0.3 ^bc^	0.055 ± 0.004 ^a^

* BC/Col/TG_WM15_ and BC/Col/TG_WM120_–BC/Col cross-linked with TG_WM_ for 15 and 120 min, respect.ively; BC/Col/TG_GS15_, and BC/Col/TG_GS120_–BC/Col cross-linked with TG_GS_ for 15 and 120 min, respectively. **^,^ *** Mean value of 14 and 3 measurements, respectively, ±standard deviation; the values in the columns marked with various letters (a–c) differ significantly (*p* < 0.05).

## Data Availability

The data presented in this study are available on request from the corresponding author.

## Abbreviations

BC.	Bacterial cellulose
Col	Collagen
CrI	Crystallinity index
DTG	Differential thermogravimetric curve
EDC	1-Ethyl-3-(3-dimethylaminopropyl) carbodiimide
FT-IR	Fourier transform infrared spectroscopy
oxBC	Oxidized bacterial cellulose
RH	Relative humidity
SEM	Scanning electron microscopy
TG	Thermogravimetric curve
TGase	Transglutaminase
TG_GS_	Transglutaminase dedicated for the fish industry
TG_WM_	Transglutaminase proposed for the meat industry
XRD	X-ray diffraction
WVP	Water Vapor Permeability
ε	Elongation at break
σ	Tensile strength
